# Life-Threatening Manifestation of Diarrhea

**DOI:** 10.1155/2013/354697

**Published:** 2013-03-27

**Authors:** Haritha Chelimilla, David Widjaja, Simeon Carvajal, Kavitha Kumbum

**Affiliations:** Division of Gastroenterology, Department of Medicine, Bronx Lebanon Hospital Center, Albert Einstein College of Medicine, 1650 Grand Concourse, 3rd Floor, Bronx, NY 10457, USA

## Abstract

Acute diarrhea is usually mild and self-limited in normally healthy individuals. Further diagnostic evaluation is indicated in patients with severe diarrhea. We present a case of a 46-year-old woman who presented with dehydration and acute renal failure due to acute severe diarrhea. Initial stool study was suggestive of *Aeromonas*. Further stool test revealed offending bacteria, which led to appropriate antibiotic use. This paper emphasizes the importance of complete history and correlation between clinical data and laboratory tests.

## 1. Introduction

Cholera is an acute intestinal infectious disease caused by gram-negative bacterium *Vibrio cholerae* and has variable manifestations. It has been responsible for several pandemics and is endemic in over 50 countries. In 2011, a total of 58 countries from all continents reported cholera cases to World Health Organization (WHO). The total number of cases reported in 2011 worldwide was 5,89,894 with total deaths 7816 [1]. It is estimated that at least 3–5 million persons develop clinical cholera each year, resulting in approximately 100,000 to 120,000 deaths [2]. In the United States, incidence of cholera is very low (0–5 cases per year) primarily due to ingestion of contaminated food, and about half of these cases are imported [3]. The most recent outbreak was reported in Haiti in 2010. Early recognition based on the history and clinical features is vital. More than 80% of cholera cases respond to oral rehydration therapy. If left untreated, 25–50% of typical cholera cases are fatal [3].

## 2. Case Report

**Figure 1:**
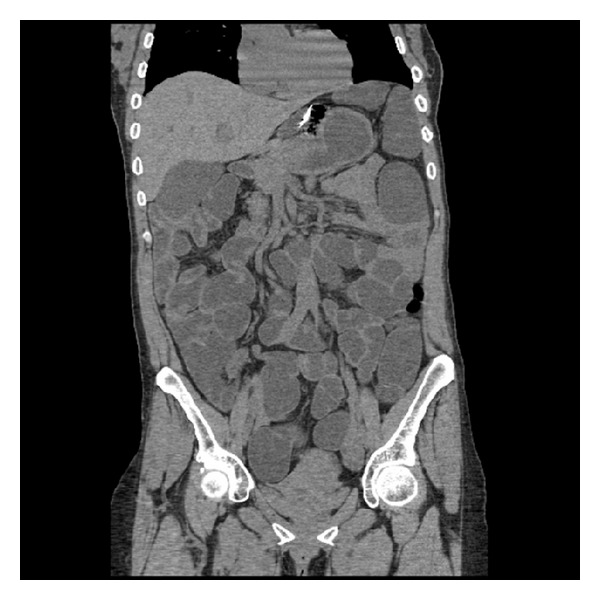
Computed tomography abdomen image showing nondilated bowel loops.

A 46-year-old Dominican woman presented to emergency room with complaints of five hours of vomiting and profuse diarrhea in summer 2011. Symptoms started within 3 hours after eating chicken sandwich and salad, which were prepared at home. While frequency of vomiting decreased, she reported diarrhea, which later occurred and was too often to count. Subsequently, her urine output decreased. The patient denied fever or blood in the stool. She had previously been healthy and was not taking any medications. She had no known allergies. Her last travel to Dominican Republic or outside the US was more than 3 years ago. She lived with her husband and son. She consumed alcohol occasionally and did not smoke or use illicit drugs. Her family and friends who had the dinner together had been well. She had no history of similar symptoms in the past. She denied recent use of antibiotics or hospitalization. She had gastric bypass surgery in 2005 and abdominoplasty in 2007. At presentation, she was afebrile with pulse rate of 103 beats/minute. Her blood pressure was 89/52 with orthostatic changes. Her mucous membranes were dry; the remainder of the examination was unremarkable. Laboratory test results were significant for anionic gap metabolic acidosis with acute kidney injury and severe hypokalemia. There was no leucocytosis. Liver enzyme tests showed mildly increased aminotransferases. The laboratory data at presentation are presented in Table 1. The patient was admitted to the critical care unit due to acute renal failure with severe electrolyte abnormalities. Human Immunodeficiency Virus test was negative. She had central line placement for close monitoring of central venous pressure. She had a total stool output of 40 liters by the end of 5 days. Later, her stool color changed to green. Stool ova and parasites and *Clostridium difficile* toxin were negative. Computed tomography scan of abdomen showed normal bowel pattern without any dilated loops of bowel and unremarkable pancreas (Figure 1). She later recalled eating cheese brought by relatives from Dominican Republic 1 day prior to the onset and at the dinner, which occurred 3 hours prior to the onset of diarrhea. Initial stool cultures were suggestive of Aeromonas; therefore, the patient was started on trimethoprim/sulfamethoxazole. Despite antibiotic treatment, there was no significant improvement of her diarrhea. Later, identification of organism from stool culture showed Vibrio cholerae O1 serovar ogawa strain. The Vibrio cholerae was resistant to trimethoprim/sulfamethoxazole; therefore, the antibiotic was changed to ciprofloxacin. Diarrhea resolved in 5 days with normalization of her renal parameters. She received 3 units of blood transfusion in view of her anemia. She developed abnormal liver function tests during the hospitalization and work-up revealed unremarkable hepatitis serology. The cause of the deranged liver function tests was probably secondary to medications and subsequently improved at the time of discharge. The patient was discharged after 7 days of hospitalization. Her caregivers were instructed about standard precautions. The case was reported to the New York State Department of Health and the US Centers for Disease Control and Prevention.

## 3. Discussion

Cholera should be considered in all cases of severe watery diarrhea and vomiting, especially those with rapid and severe dehydration, even in the absence of travel history. *Vibrio cholerae* are acid-sensitive organisms. The incubation period of cholera is variable from a few hours to five days. Incubation period is shortest among patients who have a high gastric pH and a high inoculum of ingested organisms [4, 5]. Gastric bypass surgery decreases bacterial acid exposure; therefore, a high number of bacteria are able to pass gastric barrier. This may explain why our patient's family members did not experience any symptoms. In 1885, Robert Koch demonstrated that guinea pigs could easily be infected with cholera if that organism was given with bicarbonate [6]. Gitelson [7] also reported a significant association between cholera and gastric acidity. He observed that five out of 25 patients with cholera in Jerusalem had undergone subtotal gastrectomy or vagotomy and pyloroplasty. Hypochlorhydria is known to increase the risk of infection by *Salmonella* sp., *Shigella* sp., *Brucella* sp., *Escherichia coli*, and *Vibrio cholerae* as well as parasites such as *Giardia* sp. [8, 9]. Neutralization of gastric acid by histamine 2 receptor antagonists (H2RAs) reduces the minimal infecting inoculum of Vibrio cholera and also increases the incidence and severity of cholera [8, 10, 11]. Perhaps this explains the severe manifestation of the infection in our patient who had altered gastric anatomy and lack of gastric acid secondary to gastric bypass surgery. In addition, decreased gastric acid state may lead to iron malabsorption, which may explain the presence of iron deficiency anemia in this patient. 

Cholera is most commonly associated with seafood. Water has been the primary vehicle for transmission and source of cholera. Drinking untreated river water was reported to be associated with the 2010 cholera outbreak in Haiti and Dominican Republic [12]. Cholera infection can also be associated with eating contaminated food [13]. Food items associating with outbreaks are fruits, vegetables, meat, cooked grains, and so forth, which are usually contaminated by contact with water or through improper handling by infected persons [13]. Our patient denied any seafood intake but the role of cheese as a possible harbinger of the bacteria remains a possibility, though cheese has been more commonly implicated in the outbreaks of *Listeria* and *E. coli* gastroenteritis [14].

Treatment should be started before identification of the causative organism as dehydration can be life threatening. Diagnostic work-up consists of Gram stain or culture of stool specimens, which are obtained at the onset of diarrhea with detection of toxinogenic strains. In our patient, initial gram stain characteristics were suggestive of Aeromonas species. Aeromonas species have been associated with wide range of diarrheal illnesses including choleric diarrhea with rice water stools [15]. Aeromonas and Vibrio cholerae are commonly misidentified. Both V. cholerae and Aeromonas spp. are oxidase positive, polar flagellated gram negative rods. Vibrio species are susceptible to the compound O/129 (2,4-diamino-6,7-diisopropylpteridine phosphate), which differentiates them from Aeromonas species, which are resistant to O/129. In addition, Vibrio grows on media containing 6% sodium chloride solution, whereas Aeromonas does not [16].

The primary goal of treatment is focused on correction of fluid and electrolyte abnormalities. Assessment of severity of hypovolemia is crucial to guide fluid replacement. Antibiotic therapy has an adjunct role to appropriate rehydration. With effective antibiotic therapy, the frequency of diarrhea and duration of illness are shortened by about 50% [17, 18]. Regional antibiotic susceptibility patterns should be used to guide the choice of therapy [19]. Currently, the epidemic strain in Haiti is susceptible to tetracycline and azithromycin; however, it is resistant to nalidixic acid, sulfisoxazole, and trimethoprim/sulfamethoxazole [18]. Our patient was initially started on trimethoprim/sulfamethoxazole due to initial diagnosis of Aeromonas. Subsequently, the culture revealed Vibrio cholera, which was resistant to trimethoprim/sulfamethoxazole but susceptible to ciprofloxacin. Therefore, the antibiotic was changed accordingly. This case illustrates the severity of cholerae in patients who have decreased gastric acid secretion, especially due to gastric resection and acid reducing agents. It also emphasizes the importance of obtaining history of ingestion of imported foods.

## Figures and Tables

**Table 1:** Laboratory test results.

Laboratory values	Lab results on admission	Lab results on day 1	Lab results on day 7	Normal values
Hematocrit	31.4	24.7%	33.5	42–51%
White blood cell (WBC) count	12.8	8.5	8.4	4.8–10.8 K/uL
Platelet count	555 k	346 K	403 k	150–400 K/uL
Prothrombin time	11.9	—	—	9.5–12 seconds
Activated partial thromboplastin time	27.4	—	—	26.1–33.8 seconds
Serum sodium	132	135	139	135–145 mEq/L
Serum potassium	3.6	2.6	3.6	3.5–5 mEq/L
Serum chloride	98	106	102	98–108 mEq/L
Serum blood urea nitrogen (BUN)	33	37	4	6–20 mg/dL
Serum creatinine	2.7	5.4	0.6	0.5–1.5 mg/dL
Serum bicarbonate	9	8	32	24–30 mEq/L
Anion gap	25	21	5	9–15 mmoles/L
Serum lipase	105	—	—	<61 U/L
Serum total protein	10.3	7.2	6.2	6–8.5 g/dL
Serum albumin	5.7	4.2	3.1	3.2–4.8 g/dL
Serum alanine aminotransferase	23	18	162	5–40 U/L
Serum aspartate transaminase	41	57	118	6–36 U/L
Serum alkaline phosphatase	70	62	256	42–98 U/L
Serum total bilirubin	0.4	0.1	0.2	0.2–1.2 mg/dL
Serum ferritin	—	9.1	—	30–400 ng/mL
